# Diagnostic Challenges and Pathogenetic Differences in Biomass-Smoke-Induced versus Tobacco-Smoke-Induced COPD: A Comparative Review

**DOI:** 10.3390/diagnostics14192154

**Published:** 2024-09-27

**Authors:** Joytri Dutta, Sabita Singh, Mandya V. Greeshma, Padukudru Anand Mahesh, Ulaganathan Mabalirajan

**Affiliations:** 1 Molecular Pathobiology of Respiratory Diseases, Cell Biology and Physiology Division, Council of Scientific and Industrial Research (CSIR)-Indian Institute of Chemical Biology (IICB), Kolkata 700091, WB, India; joytridutta@gmail.com (J.D.); ssabita26@gmail.com (S.S.); 2 Academy of Scientific and Innovative Research (AcSIR), Sector-19, Kamla Nehru Nagar, Ghaziabad 201002, UP, India; 3 Department of Respiratory Medicine, JSS Medical College, JSS Academy of Higher Education & Research, Mysuru 570015, KA, India; greeshmagreekz27@gmail.com (M.V.G.); mahesh1971in@gmail.com (P.A.M.)

**Keywords:** COPD diagnosis, biomass smoke, BSCOPD, TSCOPD, oxidative stress

## Abstract

**Background:** Chronic Obstructive Pulmonary Disease (COPD) is a major global health challenge, primarily driven by exposures to tobacco smoke and biomass smoke. While Tobacco-Smoke-Induced COPD (TSCOPD) has been extensively studied, the diagnostic challenges and distinct pathogenesis of Biomass-Smoke-Induced COPD (BSCOPD), particularly in low- and middle-income countries, remain underexplored. **Objective:** To explore the differences in clinical manifestations, pulmonary function, and inflammatory profiles between BSCOPD and TSCOPD and highlight the diagnostic complexities of BSCOPD. **Methods:** This review analyzes the current literature comparing BSCOPD with TSCOPD, focusing on distinctive pathophysiological mechanisms, inflammatory markers, and oxidative stress processes. **Results:** BSCOPD presents differences in clinical presentation, with less emphysema, smaller airway damage, and higher rates of pulmonary hypertension compared to TSCOPD. BSCOPD is also characterized by bronchial hyperresponsiveness and significant hypoxemia, unlike TSCOPD, which exhibits severe airflow obstruction and emphysema. Additionally, the inflammatory profile of BSCOPD includes distinct mucous hypersecretion and airway remodeling. **Conclusions:** The unique genetic, epigenetic, and oxidative stress mechanisms involved in BSCOPD complicate its diagnosis and management. Biomass smoke’s underrecognized impact on accelerated lung aging and exacerbation mechanisms emphasizes the need for targeted research to refine diagnostic criteria and management strategies for BSCOPD. **Future directions**: Further research should focus on identifying specific biomarkers and molecular pathways to enhance early diagnosis and improve clinical outcomes in populations exposed to biomass smoke.

## 1. Introduction

Chronic Obstructive Pulmonary Disease (COPD) is a progressive, life-threatening lung disease that claimed about 3 million lives globally in 2019, making it the third-leading cause of death worldwide [1]. COPD is characterized by persistent respiratory symptoms and airflow limitation due to airway and/or alveolar abnormalities caused by significant exposure to noxious particles or gases, with host factors such as abnormal lung development also playing a role [2]. Common symptoms include difficulty breathing, chronic cough, wheezing, chest tightness, and sputum production. COPD has been classified based on the extent of airflow limitation as a percentage of the normal value of forced expiratory volume in 1s (FEV1) by GOLD as mild (GOLD1, FEV1: greater than 80% of the predicted value), moderate (GOLD 2, FEV1 79–50% of the predicted value), severe (GOLD3, FEV1: 49–30% of the predicted value), and very severe (GOLD 4, FEV1 less than 30% of the predicted value) [2].

Chronic inflammation and fibrosis in COPD lead to fixed narrowing of the bronchiolar periphery, disruption of alveolar attachments due to emphysema, and airway occlusion with mucus and inflammatory exudates, resulting in irreversible airflow limitation characterized by an FEV1/FVC ratio < 0.7 and FEV1 < 80% predicted. This airflow limitation causes gas trapping, hyperinflation, and reduced inspiratory capacity, leading to shortness of breath on exertion [2]. Risk factors for COPD include cigarette smoke, biomass fuel smoke, occupational dust and fumes, and both indoor and outdoor air pollution [2]. A study found that dust exposure and smoking significantly increase COPD risk among coal mine workers, with a prevalence of 22.66%, but no interaction between these risk factors was observed [3]. Another pioneering study in the Netherlands investigated the link between living near intensive animal farming and respiratory mortality. Their findings suggested that proximity to pig farms correlated with higher respiratory disease mortality, particularly COPD and pneumonia [4]. While cigarette smoking is the primary cause, only 20% of smokers develop COPD, suggesting additional host factors such as genetic predispositions and accelerated aging. Over 90% of COPD-related deaths occur in middle- and low-income countries, where high exposure to biomass smoke is prevalent [5]. In high-income countries, tobacco smoking accounts for over 70% of COPD cases, while low- and middle-income countries experience 30–40% of COPD cases related to tobacco use, with significant proportions attributed to indoor air pollution from biomass fuels and vehicular emissions [6].

### Biomass-Smoke-Induced COPD

Historically, Homo erectus used wood fires for cooking and heating. Biomass fuels, including wood, charcoal, dried twigs, crop wastes, and animal dung, have been used throughout history. Despite modern cleaner alternatives, many rural households still rely on biomass fuels [7]. In 2010, exposure to solid-fuel use accounted for 3.2 million deaths and 111 million disability-adjusted life-years worldwide [8]. Because the exposure is primarily domestic, it most commonly involves women and infants; nevertheless, males can also be affected, especially in cases when biomass is utilized for residential heating. The geographic isolation of a population and poor availability of cleaner fuels are reasons for using biomass fuel, creating a low position on the energy or fuel ladder. Biomass fuels are inefficient and produce high levels of pollutants such as carbon monoxide (CO), polycyclic aromatic hydrocarbons (PAHs), fine particulate matter (PM10), aldehydes, benzene, nitrogen oxides, sulfur oxides, and free radicals [9]. PM10 levels in biomass-fuel households can be 10–70 times higher than ambient levels, and CO levels can reach up to 500 ppm during cooking, far exceeding safety thresholds [7].

Women, young girls, and children are particularly vulnerable due to prolonged exposure during cooking in poorly ventilated environments. In developing countries, girls start cooking around the age of 15 and spend 4 to 6 h daily in kitchens, leading to significant biomass smoke exposure [10]. Prolonged exposure has been linked to respiratory conditions, including COPD, due to carbon particle deposition and other pollutants in the airway mucosa, causing long-term inflammatory changes [11].

Biomass-smoke-induced COPD differs from cigarette-smoke-induced COPD. It results in higher lung diffusion capacities for carbon monoxide (DLCO) and milder airflow obstruction, but also thicker pulmonary arterial intima, greater pigment accumulation, and fibrosis, collectively referred to as bronchial anthracofibrosis. Biomass-smoke-induced COPD shows less emphysema and epithelial damage, increased basement membrane thickening, and air trapping compared to cigarette smoke exposure. The lymphocytic-predominant airway inflammation observed in biomass smoke COPD contrasts with the decreased macrophages and increased neutrophils in cigarette smoke COPD, reflecting variations in immune responses to different smoke components [12]. Understanding these mechanisms is crucial for addressing COPD effectively, given the significant global health impact of biomass smoke exposure.

## 2. Comparative Analysis of Biomass-Smoke-Induced and Tobacco-Smoke-Induced COPD: Clinical Profiles, Pulmonary Function, and Public Health Implications (Figure 1)

Chronic Obstructive Pulmonary Disease (COPD) resulting from biomass smoke (BSCOPD) and tobacco smoke (TSCOPD) exhibits unique pathological characteristics, clinical manifestations, inflammatory responses, and demographic patterns despite sharing some common respiratory symptoms. Acknowledging these variations is imperative for precise diagnosis and tailored treatment strategies. From a demographic standpoint, BSCOPD primarily impacts women from rural settings who have had lifelong exposure to biomass smoke [13]. This encompasses individuals who were exposed to biomass smoke during prenatal childhood stages, which adversely influences lung development and augments the susceptibility to airway damage and disease in subsequent years [14].

**Figure 1:**
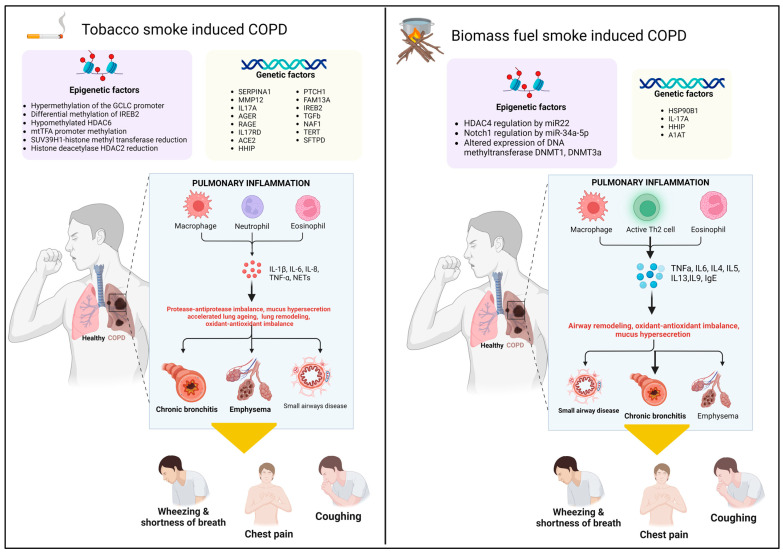
Comparative analysis of Tobacco smoke (TS)-induced COPD and Biomass smoke (BS)-induced COPD in terms of genetic and epigenetic factors, inflammation, and pathological features. Epigenetic alterations increase the risk of developing TSCOPD or BSCOPD. Gene polymorphism also increases the susceptibility to develop TSCOPD or BSCOPD. In TSCOPD, inflammatory cells like macrophages, neutrophils, and eosinophils release inflammatory cytokines such as IL-1β, IL6, IL8, etc., leading to protease–antiprotease imbalance, mucus hypersecretion, accelerated lung aging, lung remodeling, and oxidative stress. This results in pathophysiological features like chronic bronchitis, emphysema, and small airway disease, which are manifested as clinical features like wheezing, shortness of breath, chest pain, and chronic cough. In BSCOPD, inflammatory cells like macrophages, eosinophils, T helper 2 cells, etc., secrete inflammatory mediators like TNFα, IL6, IL4, IL5, IL13, IgE, etc., which ultimately leads to airway remodeling, oxidative stress, and mucus hypersecretion. These pathophysiological features manifest as clinical symptoms like wheezing, shortness of breath, chest pain, and chronic cough.

Demographically, women and their children are the most vulnerable population primarily due to the cooking duties foisted on women within inadequately ventilated kitchens [15]. Conversely, TSCOPD is more prevalent in individuals with a substantial history of heavy smoking, which often begins in adolescence and is especially common in urban dwellers. Women diagnosed with BSCOPD exhibit shorter stature, higher body mass index (BMI), and are older, implying they may need a longer duration to manifest the disease [16]. BSCOPD is pathologically distinguished by a higher occurrence of anthracosis [17], small airway fibrosis, intimal thickening in small pulmonary arterioles, and mosaic perfusion pattern as opposed to TSCOPD [18,19], where goblet cell hyperplasia is more frequently observed. Autopsy examination in individuals with BSCOPD demonstrates increased intimal thickening of the pulmonary artery small vessels, potentially manifesting into pulmonary hypertension, alongside airway disease and a lesser extent of emphysema than is typically seen in TSCOPD [20]. In addition to these pathological changes, various radiological differences have been found between TSCOPD and BSCOPD [21,22,23].

When assessing the disparity in clinical symptoms, BSCOPD exhibits a prevalence of chronic cough and phlegm production [24,25], and airway thickening, with dyspnea also being frequently documented. These symptoms are also seen in TSCOPD but may be less prominent. Airway hyperresponsiveness (AHR) is commonly encountered in BSCOPD [26] while wheezing in TSCOPD is often linked to chronic obstructive asthma [27]. Physical examinations in individuals with BSCOPD uncover low-pitched crackles, while chest roentgenogram exhibits normal findings or demonstrates increased bronchovascular markings [28]. Physical examination of TSCOPD may indicate wheezing, prolonged expiration, and hyperinflated lungs.

Table 1 highlights the key radiological differences between Tobacco-Smoke-Induced COPD (TSCOPD) and Biomass-Smoke-Induced COPD (BSCOPD), particularly from wood smoke exposure. TSCOPD is characterized by a predominance of emphysema, with larger lung volumes and more significant emphysematous changes. In contrast, BSCOPD shows an airway-predominant phenotype, with prominent bronchial wall thickening, bronchiectasis, and mosaic perfusion patterns. These radiological distinctions underscore the diverse pathophysiological impacts of different COPD etiologies, emphasizing the need for tailored diagnostic and therapeutic approaches.

With reference to pulmonary function, BSCOPD has less obstruction of airflow in comparison to people with TSCOPD who experience severe obstructions in airflow with frequent exacerbations. The rate of decline in FEV1 is also slower in BSCOPD [29]. Because of the lower severity of emphysema in BSCOPD, the diffusing capacity of the lungs for carbon monoxide (DLCO) is also rarely low, contrary to TSCOPD, where emphysema is frequent and severe and evident on imaging with high-resolution computed tomography, and, therefore, the DLCO is often reduced [21]. Pulmonary artery changes, which lead to pulmonary hypertension and cor pulmonale due to hypoxemia, are more frequent in BSCOPD [30]. That said, severe cases with pronounced emphysema in TSCOPD can still suffer from cor pulmonale, though this is less common. Research has demonstrated higher methacholine hyperresponsiveness in patients with BSCOPD, which correlates to the asthma–COPD overlap phenotype, than in patients with TSCOPD [31]. Differences in lung function include more significant airflow obstruction in TSCOPD and restrictive ventilatory alterations in BSCOPD. BSCOPD patients also have higher airway resistance and lower conductance than patients with TSCOPD [7].

Hypoxemia is more prevalent in individuals with BSCOPD, which contributes to the onset of pulmonary hypertension [32]. Conversely, TSCOPD displays a varied hypoxemic state often knitted with the severity of emphysema and chronic bronchitis. Tomography and histological evidence reveal that BSCOPD is characterized by a lower degree of emphysema and greater incidences of airway alterations that are inclusive of thickening of bronchial and peri-bronchial tissues, fibrosis, and accumulation of anthracotic pigment [23,28].

Recent studies have substantiated a noteworthy disparity in the quality of life for females suffering from BSCOPD compared to those suffering from TSCOPD. Camp et al. concluded in 2014 that women with BSCOPD endured severe symptoms and also had substantial impediments in performing everyday activities compared to TSCOPD patients [23]. In a related study, González-García et al. conducted a study among 138 COPD women and concluded that patients affected with BSCOPD had a considerably lower quality of life and greater dyspnea than those with TSCOPD, despite the former group having an identical degree of airflow obstruction [26].

From a public health perspective, BSCOPD primarily affects women and children in disadvantaged, rural areas, necessitating interventions like improved vented biomass stoves to reduce exposure [33]. TSCOPD affects a broader demographic, requiring anti-smoking campaigns and policies to reduce tobacco use. Survival rates and exacerbation frequencies are similar between the two groups after adjusting for variables like age, sex, and disease severity.

In summary, BSCOPD and TSCOPD differ significantly in terms of demographics, severity of airflow obstruction, and emphysema. Pulmonary artery changes and pulmonary hypertension are more common in BSCOPD, while clinical symptoms of cough, phlegm, and airway thickening are more pronounced. TSCOPD is characterized by more severe emphysema and wheezing. Understanding these differences is essential for developing targeted interventions and improving the clinical management of COPD patients based on their specific exposure history.

## 3. Genetic and Epigenetic Factors in COPD Pathogenesis: Cigarette Smoke vs. Biomass Smoke

### 3.1. Genetic Factors

Genetic factors (Table 2) play a significant role in COPD development, influencing susceptibility beyond environmental exposures. For instance, the *SERPINA1* gene, located on chromosome 14 and encoding α1-antitrypsin, is a prominent genetic risk factor. Its deficiency, due to homozygosity of the Z allele, leads to unchecked neutrophil elastase activity and lung damage, contributing to emphysema [34]. Similarly, polymorphisms in *MMP12*, an enzyme degrading elastin, are associated with increased susceptibility to emphysema and COPD [34].

The *AGER* gene, which encodes the receptor for advanced glycation end products (RAGE), is implicated in COPD through its role in neutrophil recruitment and elastolysis. Elevated RAGE levels in COPD lungs serve as a biomarker for disease severity [35]. Additionally, IL-17A and its receptor IL-17RD are associated with chronic inflammation in COPD, mediating neutrophilia [36,37]. ACE2, the receptor for SARS-CoV-2, is upregulated in COPD patients, suggesting a genetic predisposition to severe COVID-19 outcomes [38]. Fricke-Galindo et al. found that BSCOPD patients with the rs3134940-TC genotype had lower sRAGE levels. While no significant association with TSCOPD was found, sRAGE levels correlated with the condition [39].The *HHIP* gene, regulating the hedgehog pathway essential for lung development, is consistently identified in GWAS studies as protective against COPD [37]. Variants in *FAM13A* affect the Wnt/β-catenin pathway and promote fatty acid oxidation, linking it to COPD [37]. *IREB2*, involved in iron metabolism, impacts COPD susceptibility by influencing mitochondrial iron loading [37]. The TGFβ pathway, particularly TGFβ2, is also implicated in COPD, although its role is less studied compared to TGFβ1 [37]. Additionally, mutations in *NAF1*, *TERT*, and *TR*, associated with telomere shortening, are linked to early-onset emphysema [40]. SFTPD, identified through GWAS, is associated with COPD risk even in non-smokers [37,41].

In biomass-smoke-induced COPD, recent research highlights several genetic factors specific to populations exposed to biomass smoke. Variants of the HSP90B1 gene, such as rs2070908, confer a decreased risk of COPD [42]. Polymorphisms in the *IL-17A* gene, including rs2275913 and rs8193036, are linked to an increased risk, underscoring the gene’s role in chronic inflammation [43]. The *HHIP* gene, known for its protective effects against COPD, shows specific SNPs like rs13118928 and rs1828591 that are significant in biomass smoke exposure [44]. *Alpha-1 antitrypsin* (A1AT) polymorphisms, such as PiS (rs17580), are notable in relation to biomass smoke exposure, though severe A1AT deficiency is rare in these populations [45]. TNF gene polymorphisms, while associated with smoking-related COPD, do not show significant associations with biomass-smoke-induced COPD [46].

### 3.2. Epigenetic Factors

Epigenetic modifications, including DNA methylation, histone modification, and non-coding RNAs, also significantly impact COPD pathogenesis. DNA methylation can repress gene expression by hindering transcription factor binding and histone interaction [47]. For example, hypermethylation of the Glutamate-cysteine ligase gene, involved in glutathione synthesis, is observed in COPD patients, resulting in decreased glutathione levels in the lungs and plasma [48]. Although cigarette smoke induces DNA methylation, only 20% of smokers develop COPD, highlighting the need to identify specific methylation patterns associated with the disease [34]. Epigenetic alterations are also present in non-smokers with COPD, with unique CpG sites identified [49], and differential methylation of the IREB2 gene has been observed independent of smoking [50].

**Table 2:** Summary of genetic factors associated with COPD pathogenesis in tobacco-smoke-induced versus biomass-smoke-induced conditions.

Gene	Function/Role in COPD	Association with Tobacco-Smoke-Induced COPD	Association with Biomass-Smoke-Induced COPD
SERPINA1	Encodes α1-antitrypsin, inhibits neutrophil elastase	Deficiency leads to increased risk of emphysema, especially with Z allele [34]	Polymorphisms like PiS (rs17580) noted, severe deficiency less common [45]
MMP12	Degrades elastin, contributing to lung tissue remodeling	Polymorphisms linked to increased susceptibility to emphysema [34]	Not specifically studied in biomass-exposed populations
AGER	Encodes receptor for advanced glycation end products (RAGE)	Elevated levels correlate with disease severity and neutrophil recruitment [35]	Not specifically studied in biomass-exposed populations
IL-17A	Mediates chronic inflammation and neutrophilia	Associated with inflammation and COPD [36,37]	Polymorphisms (rs2275913, rs8193036) linked to increased risk [43]
ACE2	Receptor for SARS-CoV-2, involved in lung pathology	Upregulated in COPD, suggesting higher COVID-19 susceptibility [38]	The rs3134940-TC genotype of ACE2 had lower sRAGE levels [39]
HHIP	Regulates hedgehog signaling, critical for lung development	Protective variants identified, linked to decreased risk [37]	SNPs like rs13118928 and rs1828591 significant in biomass smoke exposure [44]
FAM13A	Affects Wnt/β-catenin signaling and fatty acid oxidation	Variants linked to COPD susceptibility [37]	Not specifically studied in biomass-exposed populations
IREB2	Involved in iron metabolism, influencing mitochondrial function	Differential methylation observed, independent of smoking [50]	Not specifically studied in biomass-exposed populations
TGFβ2	Part of the TGFβ pathway, involved in lung tissue repair and inflammation	Less studied in COPD compared to TGFβ1, but implicated [37]	Not specifically studied in biomass-exposed populations
NAF1, TERT, TR	Involved in telomere maintenance, linked to early-onset emphysema	Mutations associated with telomere shortening and early COPD onset [40]	Not specifically studied in biomass-exposed populations
SFTPD	Surfactant protein D, involved in lung immune response	Associated with COPD risk even in non-smokers [37,41]	Not specifically studied in biomass-exposed populations
HSP90B1	Heat shock protein, involved in protein folding and stress response	Not specifically studied in tobacco smoke COPD	Variant rs2070908 associated with decreased risk of COPD [42]
TNF	Encodes TNF-α, a pro-inflammatory cytokine	Polymorphisms associated with smoking-related COPD [46]	No significant association observed with biomass-smoke-induced COPD [46]

Cigarette smoke influences COPD through various epigenetic mechanisms, such as oxidative-stress-induced ciliophagy mediated by overexpressed and hypomethylated histone deacetylase 6 (HDAC6) [51]. It also reduces mitochondrial transcription factor A expression by methylating its promoter [52]. Additionally, histone modifications play a role, with reduced levels of histone methyltransferase SUV39H1 contributing to abnormal inflammation [53]. HDAC2 is crucial for regulating inflammation by deacetylating nuclear glucocorticoid receptors. Cigarette smoke decreases HDAC2 levels through various mechanisms, including peroxynitrite-induced nitration, PI3K pathway activation, and miR223-induced degradation driven by inflammatory mediators like IL-1β and TNF-α [53,54].

In biomass-smoke-induced COPD, genetic research and epigenetic alterations are less well characterized. However, evidence suggests that similar epigenetic mechanisms may be at play. Variations in the IL-17A gene and the HHIP gene show correlations with disease risk, which could be influenced by epigenetic changes due to biomass smoke exposure [44,55]. Further research is needed to explore how DNA methylation and histone modifications might contribute to COPD pathogenesis in populations exposed to biomass smoke.

Thus, both cigarette smoke and biomass smoke exposure contribute to COPD through complex interactions between genetic and epigenetic factors. While cigarette smoke is a well-documented environmental risk factor with established epigenetic mechanisms, biomass smoke exposure in specific populations also reveals unique genetic and potential epigenetic influences on COPD susceptibility. Understanding these mechanisms is crucial for developing targeted prevention and treatment strategies for COPD.

## 4. A Comparative Analysis of Inflammatory Cells and Mediators in Tobacco Smoke and Biomass Smoke COPD

Chronic Obstructive Pulmonary Disease (COPD) is a multifaceted disease characterized by chronic inflammation of the airways and lungs (Figure 1). Exposure to irritants such as cigarette smoke, air pollutants, and biomass smoke activates pattern recognition receptors (PRRs) on macrophages and alveolar epithelial cells, initiating inflammatory responses [1]. Epithelial cells release chemotactic factors like TNF-α, IL-6, IL-1β, GM-CSF, and CXCL8 (IL-8), which recruit neutrophils to the lungs [56].

In both biomass-smoke-induced COPD (BSCOPD) and tobacco-smoke-induced COPD (TSCOPD), inflammatory mediators such as IL-1β, TNF-α, and IL-8 are elevated (Figure 1). BSCOPD is characterized by increased levels of IL-1β, TNF-α, and IL-6 in lung tissue, with higher counts of neutrophils, macrophages, and monocytes in bronchoalveolar lavage fluid (BALF). Conversely, TSCOPD features prominently high levels of IL-6, IL-8, and IL-5 [57,58].

Macrophages in COPD patients exhibit diverse phenotypes, including M1 (pro-inflammatory), M2 (anti-inflammatory), and mixed types [59]. These macrophages are involved in efferocytosis, which is defective in COPD, leading to persistent inflammation. They release neutrophil chemoattractants like LTB4, GROα, IL-8, and MCP-1, as well as IL-23, promoting Th17 cell activation and neutrophilic inflammation. Proteases such as MMP2, MMP9, MMP12, and various cathepsins, released by macrophages, contribute to emphysema [5].

Neutrophils are abundant in the blood, sputum, and BALF of COPD patients, correlating with disease severity. They produce myeloperoxidase and proteases such as NE, proteinase 3, and MMP9, which damage alveoli and stimulate mucus hypersecretion [1]. Neutrophil extracellular traps (NETs), which have antimicrobial functions, are found in excess in COPD patients and correlate with disease severity [56]. Eosinophilia, present in a subset of COPD patients, correlates with corticosteroid responsiveness [60].

Among T cells, CD8+ T cells (Tc1) predominate in COPD, releasing granzyme, perforin, and TNF-α, inducing apoptosis of alveolar epithelial cells [1]. Th17 cells are elevated relative to Treg cells in COPD, contributing to inflammation [56]. Elevated leptin levels impair T cell glycolysis and reduce Treg cell production, promoting inflammation [61]. T-cell exhaustion, marked by increased PD-1 expression, impairs cytotoxic function [62]. B cells and lymphoid follicles increase in COPD, correlating with disease severity. BAFF, a B cell maturation factor, is elevated in severe COPD [5]. Autoantibodies against ECM proteins and carbonyl-modified proteins (CMPs) are found in COPD, supporting an autoimmune component [63].

Systemic inflammation markers, including CRP, CXCL8, fibrinogen, IL-6, and TNF-α, are elevated in 70% of COPD patients, with 16% showing persistent inflammation [5]. Whether this systemic inflammation results from the “spillover” of inflammatory molecules from the lungs to the systemic circulation remains debatable.

A comparative analysis of inflammatory responses in BSCOPD and TSCOPD reveals distinct differences. Biomass smoke exposure activates pulmonary macrophages and other cells, leading to the production of IL-6, IL-8, MCP-1, MIP-2, and TNF, promoting proteolysis and tissue remodeling [64]. In contrast, TSCOPD is characterized by higher levels of IL-6, IL-8, and IL-5, and a higher frequency of Th17 cells [57,65]. BSCOPD is associated with a Th2 cytokine profile, marked by increased IL-4 and Th2 cells, whereas TSCOPD features a Th17-mediated response [58]. Understanding these differences is crucial for developing targeted therapies that address the unique inflammatory mechanisms in each type of COPD.

## 5. Eosinophilic COPD in Biomass Smoke and Tobacco Smoke: A Comparative Analysis of Inflammatory Profiles and Clinical Implications

Among individuals diagnosed with COPD, approximately 40% have eosinophilic COPD, which is an increasing phenotype of COPD characterized by a persistent eosinophilic condition in the airways and bloodstream [66]. While conventionally COPD is equated to neutrophilic inflammation, recent studies, including the ECLIPSE study (The Evaluation of COPD Longitudinally to Identify Predictive Surrogate End-points), have pointed out the key contribution of eosinophils related to risk for exacerbations and responses to therapy [67]. However, the clinical features of eosinophilic COPD are clearly different from asthma–COPD overlap syndrome. These phenotypes have been characterized in subjects without asthma by low incidences of allergies, few exacerbations, and less eosinophilic inflammation.

Several cytokines, like IL-3, IL-5, and GM-CSF, influence the differentiation and activation of eosinophils and lineage commitment. IL-5 is particularly involved in the maturation of eosinophils and the subsequent migration of these cells to sites of inflammation. In the context of eosinophilic COPD, cigarette smoke and other lung irritants provoke the airway epithelium to secrete IL-33, IL-25, and thymic stromal lymphopoietin that act to elevate the Th-2 transcriptomic signatures [68]. These cells produce IL-5, which induces eosinophil chemotaxis and chronic residency of eosinophils within the pulmonary tissue [69,70]. Eosinophils exacerbate disease pathology by secreting IL-13, which stimulates alveolar macrophages to produce matrix metalloproteinase-12 (MMP12), thereby contributing to the development of emphysema [71].

The unique inflammatory profiles of BSCOPD and TSCOPD delineate COPD’s heterogeneous nature and accentuate the role of eosinophilic inflammation in COPD (Figure 1). Biomass-smoke-related COPD is correlated with significant eosinophilia in induced sputum compared to TSCOPD. A research study conducted in Goa, India, found that 71% of BSCOPD patients had significant eosinophilia, whereas this was only the case in 49.4% of TSCOPD patients, despite all the patients having similar grades of airway limitation, disease severity, and clinical scores [72]. This finding suggests that eosinophilic inflammation may assume a more relevant role in BSCOPD.

Additionally, it has also been found that the exacerbation of eosinophilic inflammation in COPD can be aggravated by air pollution, particularly particulate matter (PM10, PM2.5) [73]. Research conducted in Taipei has demonstrated that PM10 level augmentation was associated with forced vital capacity reversibility impairment in COPD patients with eosinophilic inflammation [74]. The indications are that this phenotype is particularly susceptible to air pollution. Blood eosinophil levels have emerged as a valuable biomarker in assessing the risk of exacerbation in COPD. According to a Colombian cohort study, high blood eosinophil counts were found in patients with a history of smoking and frequent exacerbations, although the highest rate of exacerbation was reported in patients with ≥300 cells/μL eosinophil count and sputum eosinophil count of ≥3% [75]. Patients with eosinophilic chronic obstructive pulmonary disease exhibit sensitivity to inhaled corticosteroids compared to a typical COPD patient, who is characteristically corticosteroid-resistant, and a count of ≥3% is associated with a good corticosteroid response in these patients [72]. Increased responsiveness is related to high blood eosinophil counts, particularly those that surpass 300 cells/μL, which are associated with a high risk of exacerbations [76]. Eosinophils can be further induced to undergo EETosis, a form of cell death that releases eosinophil extracellular traps that may enhance the inflammatory milieu by NET formation and lead to exacerbations of pathology in COPD [77].

These observations imply that, at present, mechanisms defining eosinophilic COPD are poorly described and, therefore, there is an acute need for further investigation into the role of eosinophils in COPD pathogenesis. In line with this, the evidence of eosinophilic inflammation in COPD, particularly in view of biomass smoke exposure and air pollution, is rapidly expanding. The exact role of eosinophils in TSCOPD, along with the molecular mechanisms behind eosinophilia and its effects on lung function, requires further investigation, particularly to delineate the underlying mechanisms and improve the prognosis and management of patients with this specific COPD phenotype.

## 6. Oxidative Stress in COPD: Comparing Biomass-Smoke- and Tobacco-Smoke-Induced COPD

Chronic Obstructive Pulmonary Disease (COPD) is significantly influenced by oxidative stress, which results from both exogenous and endogenous sources of oxidants. The primary exogenous sources include air pollutants, cigarette smoke, biomass fuel smoke, and occupational dust and fumes. Understanding the similarities and differences in oxidative stress mechanisms between biomass-smoke-induced COPD (BSCOPD) and tobacco-smoke-induced COPD (TSCOPD) is crucial for targeted therapeutic approaches.

### 6.1. Cellular Responses (Figure 1)

The impact of biomass exposure on the lungs has been studied in both in vitro and in vivo conditions. The literature has established that this type of smoke increases the expression of genes encoding pro-inflammatory cytokines, such as IL-1β, TNF-α, and IL-8, in murine and human lung epithelial cells, more specifically in the BEAS-2B cell line. These cytokines play a leading role in the inflammatory response, modulating airway inflammation and related damage [11]. In a large cohort of 635 biomass-smoke-exposed women and 452 age-matched controls, biomass users showed increased serum levels of IL-6, IL-8, TNF-α, CRP, and generation of ROS, whereas SOD (superoxide dismutase) was decreased in leukocytes [78]. These findings indicate systemic inflammatory responses and elevated oxidative stress due to biomass smoke exposure. In a similar study, Falfán-Valencia et al. also reported increased systemic levels of IL-6 and IL-8. Increased levels of heme oxygenase-1 (HO-1), cytochrome P450 2E, and metallothionein-2 (MT-2) are evidence of resulting oxidative stress from biomass smoke exposure [65,79]. HO-1 is a protective enzyme induced as part of the response to oxidative stress and represents an attempted protective mechanism against cell damage [79]. However, chronic exposure can overwhelm these protective pathways and lead to an increase in cell death.

Tobacco-smoke-induced COPD (TSCOPD) is characterized by high oxidative stress due to the presence of reactive oxygen and nitrogen species, PAHs, and heavy metals like cadmium and lead [80]. The oxidants in cigarette smoke promote protein and lipid oxidation, ER stress, disrupted ceramide metabolism, and apoptosis in lung cells [81]. These oxidative processes activate DAMP receptors such as RAGE and TLRs, triggering inflammatory cascades [82]. Nrf2, a key transcription factor, is activated via oxidant-induced KEAP1 modification, enhancing lung antioxidant defenses like GSH [80,83]. However, the oxidative inactivation of DJ-1, which stabilizes Nrf2, may increase smokers’ susceptibility to COPD. Additionally, cigarette smoke causes mitochondrial membrane potential (MMP) depolarization, impairing cellular respiration and energy production, leading to ROS accumulation, oxidative damage, apoptosis, and necrosis [81].

### 6.2. Oxidative Stress Mechanisms

Both biomass-smoke-induced (BSCOPD) and tobacco-smoke-induced COPD (TSCOPD) are marked by high levels of reactive oxygen species (ROS) and reactive nitrogen species (RNS), leading to oxidative stress [84]. Cigarette smoke, rich in free radicals, contributes to epithelial injury and cell death [85]. Biomass smoke similarly generates ROS, though with differing regulatory mechanisms. Both COPD types show elevated malondialdehyde (MDA) levels, indicating lipid peroxidation, and increased superoxide dismutase (SOD) activity, correlating with reduced lung function (FEV1) [84]. However, TSCOPD exhibits higher oxidative DNA damage and more significant iron homeostasis disruption, intensifying ROS production through the Fenton reaction [85,86]. Antioxidant defenses, including glutathione and catalase, are reduced in both COPD types due to impaired Nrf2 activity, exacerbating oxidative damage [87]. Despite shared oxidative stress pathways, distinct differences in oxidative damage and inflammatory profiles between BSCOPD and TSCOPD suggest the need for tailored therapeutic approaches.

## 7. Protease–Antiprotease Imbalance in COPD: A Comparative Analysis of TSCOPD and the Emerging BSCOPD

The alveolar structure is reinforced by elastic fibers and collagen, with elastin facilitating alveolar recoil and collagen providing structural integrity. In COPD, the degradation of elastin leads to the permanent enlargement of alveolar spaces, resulting in emphysema—a hallmark of COPD. This condition affects about 20% of smokers, typically emerging after prolonged exposure. Unlike elastin, collagen increases in emphysematous lungs as a reparative response, while elastin fragmentation generates chemotactic factors that attract macrophages, leading to further tissue damage. Desmosine and isodesmosine, unique biomarkers of elastin breakdown, are elevated in the urine and blood of COPD patients [88].

The “protease-antiprotease imbalance hypothesis” posits that an excess of protease activity, or a deficit in antiprotease defenses, results in the ECM degradation seen in emphysema [89]. Key proteases implicated include neutrophil elastase (NE), MMP-9, and MMP-12. NE, predominantly produced by neutrophils, targets elastin and has been linked to COPD severity. NE is found in higher amounts in the exhaled breath condensates and saliva of COPD patients [90]. Additionally, NE transcriptionally regulates the expression of other proteases like MMP-2, MMP-9, and cathepsin B [91]. MMP-12, primarily secreted by macrophages, has elastin as its main substrate and is elevated in COPD lungs, with a specific SNP in the MMP12 gene promoter linked to COPD susceptibility [88].

Antiproteases such as alpha-1 antitrypsin (A1AT) and elafin counteract the destructive effects of proteases. However, an imbalance between proteases and their inhibitors has been shown to disrupt lung repair mechanisms, particularly in tobacco-smoke-induced COPD (TSCOPD) [91,92]. Recent studies challenge the traditional protease–antiprotease imbalance hypothesis, suggesting that certain proteases, like ADAM8, might protect against emphysema development in COPD [93]. Additionally, antiproteases may increase during COPD exacerbation, potentially as a natural immune response [94].

While much is known about this imbalance in TSCOPD, the dynamics in biomass-smoke-induced COPD (BSCOPD) remain underexplored. BSCOPD, a significant cause of COPD in low- and middle-income countries, differs from TSCOPD in its pathophysiology, with less emphysema and more prominent small airway fibrosis. These differences suggest that the protease–antiprotease imbalance in BSCOPD may diverge from that seen in TSCOPD. Understanding these dynamics will be crucial for developing targeted therapies for BSCOPD, a condition that may involve unique mechanisms of lung injury.

In conclusion, while the protease–antiprotease imbalance is well-characterized in TSCOPD, the impact of biomass smoke on this balance in BSCOPD remains an area of emerging research. Elucidating these mechanisms will be key to advancing the treatment of BSCOPD and improving outcomes for affected populations.

## 8. Mucus Hypersecretion in COPD: Tobacco Smoke vs. Biomass Smoke Exposure

The airway epithelium is typically covered by a thin mucus layer, which hydrates the airways, traps inhaled particles and microorganisms, and expels them via a cough or mucociliary clearance. Mucins, primarily MUC5AC and MUC5B, are the key glycoproteins in mucus, synthesized by goblet cells and submucosal glands. Mucin production is triggered by stimuli like cigarette smoke (CS), particulate matter, and biomass smoke, which activate receptors such as Toll-like receptors, leading to ROS production. This activates membrane-bound proteases like ADAM17, which cleave pro-ligands to activate the EGFR pathway, ultimately inducing mucin mRNA and protein synthesis [95].

In COPD, mucus hypersecretion is a significant pathological feature. CS, the primary cause of COPD, triggers mucus production through various pathways. It induces mitochondrial ROS, which promotes autophagy and increases MUC5AC expression via JNK and EGFR signaling. CS also disrupts the function of the CFTR channel, leading to increased mucus viscosity and impaired clearance [96]. Additionally, CS exposure leads to the internalization of MUC1, prolonging EGFR activation, causing goblet cell metaplasia and MUC5AC overproduction [97].

Biomass smoke exposure, a significant cause of COPD in low-income regions, also induces mucus hypersecretion. Studies show that biomass smoke increases the expression of mucin genes (MUC1, MUC5AC, MUC5B), affecting mucin homeostasis similarly to chronic bronchitis. Ciliary dysfunction is more pronounced in response to biomass smoke, suggesting that prolonged exposure disrupts normal respiratory function and increases the risk of lung diseases [98]. Unlike CS, which promotes goblet cell metaplasia, biomass smoke exposure activates mucin expression independently of cellular infiltrates, through pathways like p53 [99].

In summary, both tobacco and biomass smoke induce mucus hypersecretion in COPD, albeit through distinct mechanisms. Tobacco smoke primarily acts through oxidative stress and signaling pathways involving EGFR, while biomass smoke impacts mucin production through alternative pathways, including p53, highlighting the need for tailored therapeutic strategies for different COPD etiologies.

## 9. Airway Remodeling in Biomass Smoke vs. Tobacco Smoke COPD

Airway remodeling in COPD involves structural changes such as subepithelial fibrosis, epithelial–mesenchymal transition (EMT), basement membrane thickening, and increased smooth muscle mass, all of which contribute to airway obstruction and airflow limitation [100]. Chronic exposure to noxious agents like cigarette smoke and air pollutants induces oxidative stress that persists even after smoking cessation, driven by inflammatory cells. This sustained oxidative stress disrupts the epithelial barrier by destroying tight junctions and releases proteases that damage alveolar attachments, triggering an aberrant repair response that leads to remodeling [101].

Pro-fibrotic mediators such as TGF-β and endothelin, produced by epithelial cells and macrophages, are key drivers of airway remodeling. TGF-β stimulates extracellular matrix (ECM) production through the SMAD pathway, while endothelin activates fibroblasts via ETA receptors, promoting fibrosis [70]. EMT is driven by pathways like TGF-β, WNT, and uPAR, leading to the downregulation of epithelial markers like E-cadherin and the upregulation of mesenchymal markers such as vimentin [101]. Myofibroblasts, derived from fibroblasts or other cells, contribute to ECM deposition and remodeling [102]. Crosstalk between epithelial cells and fibroblasts amplifies this response, as seen in cigarette-smoke-induced miR-21 transfer, which promotes myofibroblast differentiation via pVHL/HIF-1α signaling [103].

The pattern of smoke inhalation varies between biomass-smoke-exposed COPD (BE-COPD) and tobacco-smoke-exposed COPD (TE-COPD). Biomass smoke, typically inhaled during regular tidal breathing, primarily damages small airways, resulting in an airway-dominant phenotype. In contrast, tobacco smoke, inhaled in two stages, penetrates deeper into the lungs, leading to an emphysema-dominant phenotype [104].

Pathological examinations of BE-COPD lungs reveal significant small airway remodeling, peribronchiolar fibrosis, and thickened bronchial walls, often accompanied by anthracotic pigment deposition [105,106]. Autopsy studies confirm that BE-COPD lungs exhibit more fibrosis and remodeling than TE-COPD lungs [107]. Radiographic findings, such as peribronchial thickening and parenchymal bands without significant emphysema, further distinguish BE-COPD from TE-COPD [108].

At the cellular level, biomass smoke exposure promotes ECM production and myofibroblast proliferation via the PI3K/AKT/TRPC1 pathway, as observed in both human and animal studies [9,109]. This evidence underscores the distinct mechanisms driving airway remodeling in different COPD phenotypes and the need for further research in this area.

## 10. Accelerated Lung Aging in COPD: Tobacco Smoke vs. Biomass Smoke

Aging leads to a decline in the body’s ability to maintain homeostasis and respond to environmental stresses, making individuals more susceptible to diseases like COPD [110,111]. The lungs, which reach maximum functional capacity around age 20–25, experience progressive damage and functional decline as part of the aging process. COPD, particularly prevalent in the elderly, is increasingly recognized as a disease of accelerated lung aging [53].

### 10.1. Tobacco-Smoke-Induced COPD (TSCOPD) and Accelerated Lung Aging

In TSCOPD, cellular senescence—a condition where cells stop dividing to prevent tumorigenesis or tissue damage—plays a critical role. This senescence can result from replicative processes, such as telomere shortening, or premature triggers like oxidative stress from cigarette smoke [112,113]. Senescence biomarkers, including β-galactosidase and p16, are elevated in TSCOPD lungs, with senescent cells secreting proinflammatory mediators known as the senescence-associated secretory phenotype (SASP) [114]. Cigarette-smoke-induced oxidative stress damages DNA, leading to markers of DNA damage like 8-OH-2dG and phosphorylated H2AX being increased in COPD patients. Impaired autophagy, telomere shortening, and associated DNA damage are additional contributors to accelerated lung aging in TSCOPD [110,112].

Furthermore, factors like autoimmunity, inflamm-aging, and epigenetic changes exacerbate lung aging in TSCOPD. Reduced levels of anti-aging proteins like SIRT1 and SIRT6, along with increased activity of pathways like mTOR and miR34a regulation, promote senescence in the lungs [112,115]. Epigenetic modifications, including DNA methylation, accelerate lung aging, with smoking shown to increase the epigenetic age of respiratory organs [114,116].

### 10.2. Biomass-Smoke-Induced COPD (BSCOPD) and the Need for Research

While much is known about accelerated lung aging in TSCOPD, the effects of biomass smoke on lung aging remain poorly understood. Biomass smoke exposure, common in many low- and middle-income countries, is a significant risk factor for COPD, yet the mechanisms by which it may accelerate lung aging have not been fully explored. Given the distinct inflammatory profiles and cellular responses between TSCOPD and BSCOPD, it is crucial to investigate whether similar pathways of cellular senescence, telomere shortening, and oxidative stress-induced DNA damage occur in BSCOPD.

Understanding the impact of biomass smoke on lung aging could reveal new therapeutic targets and prevention strategies tailored to populations exposed to this type of smoke. Research in this area is urgently needed to bridge the knowledge gap and improve outcomes for individuals with BSCOPD, who may be at risk of accelerated lung aging through mechanisms distinct from those observed in TSCOPD.

In conclusion, while TSCOPD has been well-studied in the context of accelerated lung aging, the role of biomass smoke in BSCOPD remains underexplored. Investigating these mechanisms is essential to develop targeted interventions and improve the health of individuals exposed to biomass smoke, who may face unique challenges in managing COPD related to accelerated lung aging.

## 11. Mechanism of COPD Exacerbation: A Comparative Analysis of TSCOPD and BSCOPD

COPD exacerbations, defined by the Global Initiative for Chronic Obstructive Lung Disease (GOLD) as an acute worsening of respiratory symptoms requiring a change in medication, are primarily triggered by viral and bacterial infections, environmental pollutants, and temperature fluctuations [43,117]. Exacerbations can be mild, moderate, or severe, with the latter often necessitating hospitalization. The pathogenesis of exacerbations in both Tobacco-Smoke-Induced COPD (TSCOPD) and Biomass-Smoke-Induced COPD (BSCOPD) involves complex interactions between infectious agents, the host immune response, and underlying airway inflammation.

In TSCOPD, exacerbations are predominantly driven by viral infections, particularly human rhinovirus, influenza virus, and respiratory syncytial virus, with bacterial pathogens like *Haemophilus influenzae* and *Streptococcus pneumoniae* frequently involved in secondary infections [117]. These infections exacerbate airway inflammation, increase mucus production, and impair mucociliary clearance, creating a favorable environment for bacterial growth. The role of the microbiome is significant, with exacerbations marked by an increase in taxa related to dominant pathogens and a decrease in other microbial diversity, underscoring the importance of microbiome dynamics in COPD exacerbations [118].

The impaired host response in TSCOPD is characterized by reduced antiviral cytokines and a compromised immune response, including decreased NK cell function and overexpression of ICAM-1, which facilitates viral entry into epithelial cells [43]. The inflammatory response is further exacerbated by the upregulation of the NLRP3 inflammasome and oxidative stress, leading to corticosteroid resistance and persistent airway inflammation [119,120]. Defective macrophage phagocytosis and impaired efferocytosis contribute to the failure to resolve inflammation, highlighting the complexity of immune dysfunction in TSCOPD [5].

In BSCOPD, recent studies suggest a similar risk of exacerbations compared to TSCOPD, with biomass smoke exposure leading to comparable inflammatory responses and exacerbation mechanisms [121]. Biomass smoke shares a similar composition to tobacco smoke, resulting in analogous effects on airway inflammation and exacerbation risk. Patients with long-term biomass smoke exposure have been found to have a similar incidence of moderate to severe exacerbations as those with significant tobacco smoke exposure, emphasizing the need for active treatment in BSCOPD [121].

The similarities in exacerbation mechanisms between TSCOPD and BSCOPD, including the involvement of viral and bacterial infections, environmental pollutants, and impaired host responses, suggest that both phenotypes require similar clinical management strategies. However, further research is needed to elucidate the specific pathways through which biomass smoke contributes to COPD exacerbations, particularly in regions where biomass fuel use is prevalent. Recognizing these shared mechanisms can help improve treatment approaches and patient outcomes in both TSCOPD and BSCOPD.

## 12. Treatment Recommendations for BSCOPD [16]

For patients diagnosed with BSCOPD, treatment should align with established COPD guidelines while emphasizing the importance of minimizing further exposure to biomass smoke. The standard therapeutic regimen typically includes the following:

- Bronchodilators: these are fundamental for relieving symptoms and improving lung function.
- Pulmonary rehabilitation: a comprehensive program designed to enhance physical fitness and overall quality of life.
- Oxygen therapy: essential for patients experiencing hypoxemia to ensure adequate oxygenation.
- Antibiotics: prescribed during infectious exacerbations to combat respiratory infections.
- Vaccinations: immunization against influenza and pneumococcus is highly recommended to prevent complications.

Despite these guidelines, robust scientific evidence supporting the effectiveness of these treatments specifically for BSCOPD patients is notably scarce. It is particularly concerning that such a large, vulnerable population has not been adequately represented in clinical trials, which hampers the validation of treatment protocols. In contrast, numerous studies have focused on tobacco smokers in higher-income regions, often resulting in well-established therapeutic recommendations. While existing guidelines provide a framework of interventions and medications with demonstrated efficacy, the implementation of specific protocols should consider the unique resources of and cost-effectiveness for each country. International health organizations could play a pivotal role in facilitating the development of tailored guidelines that address the needs of BSCOPD patients effectively.

## 13. Conclusions and Future Perspectives

Chronic Obstructive Pulmonary Disease (COPD) remains a critical global health issue, with its complex pathogenesis posing significant challenges for diagnosis and treatment. The disease’s heterogeneity is further highlighted by the differences between Tobacco-Smoke-Induced COPD (TSCOPD) and the less-studied Biomass-Smoke-Induced COPD (BSCOPD). While TSCOPD has been the subject of extensive research, BSCOPD—prevalent in low- and middle-income countries—presents unique diagnostic and pathogenetic challenges that have been largely overlooked.

BSCOPD differs from TSCOPD in several key aspects, including clinical manifestation, pulmonary function, and inflammatory profiles. These differences necessitate a distinct approach to diagnosis and management. BSCOPD is often characterized by less emphysema, increased bronchial hyperresponsiveness, smaller airway damage, and a higher incidence of pulmonary hypertension compared to TSCOPD. The unique genetic and epigenetic factors, along with differential oxidative stress mechanisms in BSCOPD, further complicate its diagnosis.

Given the underrecognized impact of biomass smoke on accelerated lung aging and exacerbation mechanisms, future research must prioritize the identification of specific biomarkers and pathways that can aid in the early and accurate diagnosis of BSCOPD. Understanding the role of unique inflammatory mediators, oxidative stress pathways, and cellular senescence in BSCOPD will be critical for refining diagnostic criteria. Additionally, exploring the potential of novel imaging techniques and molecular diagnostics to differentiate BSCOPD from TSCOPD could lead to more personalized treatment strategies.

In summary, addressing the complexities of COPD, particularly BSCOPD, requires a concerted effort to bridge the existing research gaps. By focusing on the distinct diagnostic challenges and pathogenetic mechanisms of BSCOPD, we can develop more effective diagnostic tools and therapeutic interventions. Such efforts are essential not only for improving clinical outcomes for patients with BSCOPD but also for reducing health disparities in regions where biomass smoke exposure is prevalent. Collaborative and targeted research in this area will ultimately contribute to a more comprehensive understanding and management of COPD on a global scale.

## Figures and Tables

**Table 1:** Radiological differences between Tobacco-Smoke-Induced COPD (TSCOPD) and Biomass-Smoke-Induced COPD (BSCOPD), including wood smoke-induced COPD.

Radiological Feature	TSCOPD	BSCOPD	References
Emphysema	Predominantly emphysema-predominant phenotype, with a higher percentage of emphysema and larger emphysematous spaces.	Less emphysema overall; when present, centrilobular or panlobular patterns observed. Emphysema is not as predominant.	[21,22]
Air Trapping	Less prominent, though present.	More air trapping, indicating an airway-predominant phenotype.	[22]
Bronchial Wall Thickening	Present, with a trend towards thicker walls.	Significant bronchial wall thickening, especially in wood-smoke-induced COPD.	[21,23]
Bronchiectasis	Less common	Common in wood-smoke-induced COPD	[22]
Mosaic Perfusion Pattern, Parenchymal Bands, Tree-in-Bud Pattern, Laminar Atelectasis	Not typically reported	observed in wood-smoke-induced COPD	[22]
Lung Volumes	Generally larger lung volumes	Smaller lung volumes	[21]
Oxygen Saturation	Less impact on oxygen saturation during rest and exercise	Lower oxygen saturation at rest and during exercise, indicating worse hypoxemia.	[22]
